# Genome-Wide Prediction of Complex Traits in Two Outcrossing Plant Species Through Deep Learning and Bayesian Regularized Neural Network

**DOI:** 10.3389/fpls.2020.593897

**Published:** 2020-11-27

**Authors:** Carlos Maldonado, Freddy Mora-Poblete, Rodrigo Iván Contreras-Soto, Sunny Ahmar, Jen-Tsung Chen, Antônio Teixeira do Amaral Júnior, Carlos Alberto Scapim

**Affiliations:** ^1^Instituto de Ciencias Agroalimentarias, Animales y Ambientales, Universidad de O’ Higgins, San Fernando, Chile; ^2^Institute of Biological Sciences, University of Talca, Talca, Chile; ^3^College of Plant Sciences and Technology, Huazhong Agricultural University, Wuhan, China; ^4^Department of Life Sciences, National University of Kaohsiung, Kaohsiung, Taiwan; ^5^Laboratory de Melhoramento Genético Veget al., Universidade Estadual do Norte Fluminense Darcy Ribeiro/CCTA, Campos dos Goytacazes, Brazil; ^6^Departamento de Agronomia, Universidade Estadual de Maringá, Maringá, Brazil

**Keywords:** deep learning, Bayesian regularized neural network, genomic prediction, machine learning, single-nucleotide polymorphisms, tropical maize, eucalypt

## Abstract

Genomic selection models were investigated to predict several complex traits in breeding populations of *Zea mays* L. and *Eucalyptus globulus* Labill. For this, the following methods of Machine Learning (ML) were implemented: (i) Deep Learning (DL) and (ii) Bayesian Regularized Neural Network (BRNN) both in combination with different hyperparameters. These ML methods were also compared with Genomic Best Linear Unbiased Prediction (GBLUP) and different Bayesian regression models [Bayes A, Bayes B, Bayes Cπ, Bayesian Ridge Regression, Bayesian LASSO, and Reproducing Kernel Hilbert Space (RKHS)]. DL models, using Rectified Linear Units (as the activation function), had higher predictive ability values, which varied from 0.27 (pilodyn penetration of 6 years old eucalypt trees) to 0.78 (flowering-related traits of maize). Moreover, the larger mini-batch size (100%) had a significantly higher predictive ability for wood-related traits than the smaller mini-batch size (10%). On the other hand, in the BRNN method, the architectures of one and two layers that used only the pureline function showed better results of prediction, with values ranging from 0.21 (pilodyn penetration) to 0.71 (flowering traits). A significant increase in the prediction ability was observed for DL in comparison with other methods of genomic prediction (Bayesian alphabet models, GBLUP, RKHS, and BRNN). Another important finding was the usefulness of DL models (through an iterative algorithm) as an SNP detection strategy for genome-wide association studies. The results of this study confirm the importance of DL for genome-wide analyses and crop/tree improvement strategies, which holds promise for accelerating breeding progress.

## Introduction

Artificial neural networks (ANNs) are computational methods of interest in the area of Machine Learning (ML) research, which has proved to be a powerful tool in several studies of genomic prediction (Drummond et al., 2003; Gianola et al., 2011; González-Recio and Forni, 2011; González-Recio et al., 2014; Leung et al., 2015; Glória et al., 2016; Romagnoni et al., 2019; Yin et al., 2019; Grinberg et al., 2020), due to its ability of dealing with a wide variety of high-dimensional problems in a computationally flexible manner (González-Recio et al., 2014; Ranganathan et al., 2018). In this regard, Gianola et al. (2011) pointed out that this method may be useful for the prediction of complex traits when the number of unknown variables is much larger than the number of samples (high-dimensional genomic information), since ANNs have the ability to capture non-linearities, adaptively (Gianola et al., 2011). Moreover, Pérez-Rodríguez et al. (2012) found that Bayesian Regularized Neural Networks (BRNNs) and Radial Basis Function Neural Networks (RBFNNs) (non-linear models) had higher predictive accuracy and smaller predictive mean-squared error than Bayesian linear regression models (Bayesian LASSO: BL, Bayesian Ridge Regression: BRR, Bayes A and Bayes B) for grain yield and days to heading in wheat.

Over the last several years, complex ANN architectures have been implemented to predict complex traits in several plant species (Ma et al., 2017; Khaki and Wang, 2019). For instance, Deep Learning (DL), a form of ML, has gained increasing interest in prediction studies, which typically use multiple hidden layers, trying to learn functions that connect the input data (inputs layer) and response variables (output layer) in absence of a model (Sheehan and Song, 2016). However, unlike traditional neural networks, algorithms of DL consider many hidden layers during the training of network (Sheehan and Song, 2016; Min et al., 2017), which transform the input data into a more abstract representation at each stacked layer (Khaki and Wang, 2019). In this regard, LeCun et al. (2015) pointed out that the use of multiple hidden layers in DL can reveal non-linear relationships between input data and response variables and can perform extremely intricate functions that are sensitive to minute details and insensitive to large irrelevant variations. Moreover, the use of non-linear functions is a powerful alternative to linear regression because it offers the most flexible curve-fitting functionality, which seeks to minimize the standard error of the estimate to increase the prediction accuracy (Abebe et al., 2018).

Rachmatia et al. (2017) implemented Deep Belief Network (DBN), one of the architectural DL methods, for developing genomic prediction models in maize and found that DBN outperformed other methods of prediction such as Reproducing Kernel Hilbert Space (RKHS), BL, and best linear unbiased predictor (BLUP). In another study, Montesinos-López et al. (2018) compared DL with the genomic BLUP (GBLUP) method using nine published genomic datasets (three of maize and six of wheat), in which the DL method had a better performance in most cases. Importantly, they corroborated that there are no universally best prediction machines, and thus, several available methods should be tested in a given breeding population. Khaki and Wang (2019) designed a deep neural network approach to predict crop yield that took advantage of state-of-the-art modeling and solution techniques. The authors pointed out that a salient feature of this learning model is that they treat the response variables as an implicit function of the input variables (e.g., genes and environmental components), which could be a non-linear function and highly complex.

In this study, genomic prediction models based on DL and BRNN were investigated to predict complex traits in two economically important plant species: *Eucalyptus globulus* Labill. and *Zea mays* L. *E. globulus* is one of the most widely planted hardwood tree species in temperate regions of the world, mostly used as raw material for pulp and paper industry due to its high-quality cellulose pulp, low lignin, and lipid content (Aumond et al., 2017). This species is also used for the production of essential oils in the pharmaceutical industry (Shao et al., 2020). Notably, *E. globulus* has been successfully grown in a broad range of environmental conditions, and it stands out as the targets of multiple breeding programs to improve economically important traits such as tree growth and wood quality (Ballesta et al., 2019). On the other hand, *Z. mays* is one of the world’s leading cereal grains along with rice and wheat (Reeves et al., 2016). This species plays an important role in food security and has many uses, including biofuel, animal feed, pharmaceutical, and agro-industrial products (Suleiman et al., 2013). Due to the importance of maize in the global context, several efforts have been undertaken addressing the efficient use of germplasm materials (Maldonado et al., 2019). Therefore, in these two important outcrossing species, we have assessed ML architectures, which were compared with GBLUP and different linear Bayesian regression models (Bayes A, Bayes B, Bayes Cπ, BRR, and BL) and the non-linear model RKHS. One of the main difficulties in implementing DL models is to find the best hyperparameter configuration, which requires time and some basic understanding of hyperparameters to optimize (Pérez-Enciso and Zingaretti, 2019; Zingaretti et al., 2020). In this regard, we evaluated the importance of mini-batch and activation function hyperparameters in terms of predictive ability (PA) and computational time required. The findings of this study can be useful as a guide to the analysis of DL for the genomic prediction of complex traits, facilitating its implementation in operational breeding programs.

## Materials and Methods

### Phenotypic and Genotypic Data

#### Maize Experiment

The panel was composed of 322 inbred lines representing a core collection of tropical maize germplasm of the State University of Maringa, Parana State, Brazil. All inbred lines were obtained from selfing and selection in nurseries, starting from populations and hybrids released by Brazilian public and private sector corn breeding programs. This maize panel was sown in the growing season 2018 in two locations of southern Brazil: Cambira and Sabaudia. The experimental design in both trials was an alpha-lattice with 24 incomplete blocks, and three replications per inbred line. The following traits related to flowering were evaluated: Male and Female Flowering time (MF and FF, respectively), measured in each line as the number of days from sowing to anther extrusion from the tassel glumes and to visible silks, respectively, and the Anthesis-Silking Interval (ASI) calculated as the difference between MF and FF (Maldonado et al., 2019). The target population of maize was genotyped using genotyping by sequencing (GBS) (Elshire et al., 2011; Glaubitz et al., 2014). The raw database of GBS was filtered considering a minor allele frequency (MAF) > 0.05, resulting in 291,633 high-quality SNPs. Subsequently, missing data were imputed through LDkNNi imputation (linkage disequilibrium k-nearest neighbor imputation) (Money et al., 2015). SNPs with a MAF < 0.01 and a proportion of missing data per location > 10% were eliminated from the imputed dataset, resulting in a total of 290,973 SNPs. Finally, a subset of 10,000 SNPs was randomly selected for genomic prediction analyses.

#### Eucalypt Experiment

This breeding population was composed of 62 full-sib and 3 half-sib families of *E. globulus* located in the La Poza sector, Purranque, administrative region of Los Lagos, Chile. The experimental design was a randomized complete block, with 30 blocks, single-tree plots, and a spacing of 2.5 m between the trees within a block. The traits were assessed in this breeding population: pilodyn penetration (WD), stem straightness (ST), branch quality (BQ), diameter at breast height (DBH), and tree height (TH). WD was indirectly estimated based on pilodyn penetration (in millimeters) at breast height using a Pilodyn 6J Forest (PROCEQ, Zurich, Switzerland) according to Valenzuela et al. (2019). ST was evaluated in the first 2/3 of the total height of the tree, considering an ordinal scale of seven levels, in which 0 represents trees with curvature in the first third of the total height of the tree, and 6 represents trees that could present a slight curvature in the upper third of the tree without loss of productivity (Ballesta et al., 2019). BQ was evaluated according to different criteria of quality (i.e., diameter, angle, and distribution of branches in the tree), considering an ordinal scale of six levels, in which 1 represents a tree with serious limitations and 6 represents a tree with all branching variables in good condition (Mora et al., 2019). Total tree height was measured using a Suunto^®^ hypsometer, while DBH was measured with a diameter tape at 1.3 m above ground level. Genomic DNA was isolated from leaf tissue of 646 individuals randomly selected from the breeding population (approximately 10 individuals per family) and genotyped using the EUChip60K SNP system (GeneSeek, Lincoln, NE, United States) (Silva-Junior et al., 2015). The genotyping quality was evaluated using Genome Studio software (Illumina, San Diego, CA, United States). Subsequently, SNPs with a MAF < 0.05 and a proportion of missing data > 10% were eliminated. A final set of 14,442 SNPs was retained.

### Heritability Estimates

Narrow-sense heritability (${\hat{h}}^{2}$) of *E. globulus* was estimated as follows:

$${\hat{h}}^{2}=\sigma_{a}^{2}/\left(\sigma_{a}^{2}+\sigma_{e}^{2}\right)$$

where $\sigma_{a}^{2}$ and $\sigma_{e}^{2}$ are the additive and residual variances, respectively. Due to the mix families (half-sib and full-sib) in *E. globulus*, only additive variance was considered. On the other hand, in *Z. mays*, broad-sense heritability (${\hat{H}}^{2}$) was computed as:

$${\hat{H}}^{2}=\sigma_{G}^{2}/\left(\sigma_{G}^{2}+\sigma_{e}^{2}\right)$$

where $\sigma_{G}^{2}$ is the total genotypic variance (i.e., $\sigma_{G}^{2}=\sigma_{a}^{2}+\sigma_{d}^{2}$; additive and dominance genetic variance, respectively). Thus, the additive and dominance ratios were estimated as:

$${\hat{h}}_{a}^{2}=\sigma_{a}^{2}/\left(\sigma_{a}^{2}+\sigma_{d}^{2}+\sigma_{e}^{2}\right)$$

$${\hat{h}}_{d}^{2}=\sigma_{d}^{2}/\left(\sigma_{a}^{2}+\sigma_{d}^{2}+\sigma_{e}^{2}\right)$$

All estimates and calculations described in this section were performed using the R package *sommer* (Team R. C., 2013; Covarrubias-Pazaran, 2016). The additive and dominance relationship matrices were estimated using the *A.mat* (Endelman, 2011) and *D.mat* (Su et al., 2012) functions, respectively.

### Genomic Prediction Models

#### Bayesian Alphabet

Genomic prediction linear Bayesian models were fitted using the following approaches: Bayesian Ridge Regression (BRR; Gianola, 2013), Bayesian Lasso (BL; Legarra et al., 2011), Bayes A (Hayes and Goddard, 2001), Bayes B (Hayes and Goddard, 2001), and Bayes Cπ (Habier et al., 2011). The models were adjusted as follows:

$$y_{i}=\mu +\sum_{j=1}^{p}A_{ij}a_{j}+\varepsilon_{i}$$

$$y_{i}=\mu +\sum_{j=1}^{p}A_{ij}a_{j}+D_{ij}d_{j}+\varepsilon_{i}$$

where *y* is a vector of phenotypes pre-corrected for non-genetic effects (i.e., block effect; experimental design); μ is an overall constant; **A**_*ij*_ and **D**_*ij*_ are genotype indicator variables for individuals *i* at locus *j*; *a_j_* and *d_j_* for *j* = 1, 2, … *p* are the additive (*a_j_*) and dominance (*d_j_*) genetic effect of the *j*th SNP; and ε_*i*_ is the residual associated to the observation on individual *i*, with distribution $\varepsilon ∼N\left(0,I\sigma_{\varepsilon }^{2}\right)$, where *I* is an identity matrix, and $\sigma_{\varepsilon }^{2}$ is the residual variance. Genotypes in **A** matrix were coded as 0 for “aa,” 1 for “Aa,” and 2 for “AA” to capture additive effects, while that for modeling dominance effects in **D** matrix, the genotypes “aa,” “Aa,” and “AA” were coded as 0, 1, and 0, respectively.

For the BRR model, the marker effect (*a_j_*) is distributed as follows: $a_{j}|\sigma_{a}^{2}∼N\left(0,\sigma_{a}^{2}\right)$, and the common variance ($\sigma_{a}^{2}$) is treated as unknown and $p\left(\sigma_{a}^{2}\right)∼\chi^{-2}\left(\sigma_{a}^{2}|df_{a},S_{a}\right)$, with degrees of freedom and scale parameter *df*_*a*_ and *S_a_*, respectively. The BL method assumes that the conditional prior distribution of each marker effect follows a double exponential (*DE*), $p\left(a_{j}|,\sigma_{\varepsilon }^{2}\right)=DE\left(a_{j}|0,\lambda ,\sigma_{\varepsilon }^{2}\right)$, where is the regularization parameter and $\sigma_{\varepsilon }^{2}$ is a specified scaled inverse Chi-squared prior density $p\left(\sigma_{\varepsilon }^{2}\right)∼\chi^{-2}\left(\sigma_{\varepsilon }^{2}|df_{\varepsilon },S_{\varepsilon }\right)$, with degrees of freedom *df*_ε_ and scale parameter *S*_ε_ (Park and Casella, 2008). Bayes A assumes that the conditional prior distribution of a marker effect *a_j_* is assumed to be Gaussian with null mean and marker-specific variance $\sigma_{a_{j}}^{2}$, independent from each other. In this model, the variance of each marker is assumed to be distributed scaled inverse Chi-squared, with $p\left(\sigma_{a_{j}}^{2}\right)=\chi^{-2}\left(\sigma_{a_{j}}^{2}|df,S^{2}\right)$, where *df* and *S*^2^ are known degrees of freedom and scale parameters, respectively (Pérez and de Los Campos, 2014). The Bayes B method assumes that only a few loci contribute with some genetic variance and that some genetic markers have zero effect, such that the prior distribution of the effects of all markers is given by:

$$\text{p}\left({\text{a}}_{j}|\sigma_{a_{j}}^{2},\pi \right)=\left\{ \begin{array}{cc} 0 & \text{w}ith\text{p}robability\pi \\ \text{N}\left(0,\sigma_{a_{j}}^{2}\right) & \text{w}ith\text{p}robability\left(1-\pi \right) \end{array}\right.$$

where π is the proportion of markers with null genetic effects. A scaled inverse Chi-square prior distribution χ^−2^(*df*_*a*_, *S*_*a*_) is assumed for $\sigma_{a_{j}}^{2}\left(j=1,\ldots ,p\right)$, which is equal for all markers (Meuwissen et al., 2001; Pérez and de Los Campos, 2014). Bayes Cπ is similar to Bayes B, in which all markers are considered to have a common variance ($\sigma_{a}^{2}$) and promote the selection of variables. The marker effects are assumed to be $a_{j}|\sigma_{a_{j}}^{2}∼N\left(0,\sigma_{a}^{2}\right)$, and the inclusion of each marker in the model is modeled by an indicator variable δ_*j*_, which is equal to 1 if the marker *j* is fitted in the model and is 0 otherwise.

The Bayesian models for maize data were implemented in the library BGLR (Pérez and de Los Campos, 2014) of R 3.6.1. All models were run with 1,000,000 iterations, a burn-in period of 100,000, and a thin of 50. For eucalypt data, the results of the Bayesian alphabet models (implemented in BGLR package) are available in Ballesta et al. (2019), which were used in this study for comparison purposes.

### Genomic Best Linear Unbiased Prediction (GBLUP) and Reproducing Kernel Hilbert Spaces (RKHS)

The GBLUP was performed using the R package *sommer* (Covarrubias-Pazaran, 2016). The additive and dominance relationship matrices were estimated using the *A.mat* (Endelman, 2011) and *D.mat* (Su et al., 2012) functions, respectively.

In the RKHS model (Gianola et al., 2006), the genomic relationship matrix used in GBLUP is replaced by a kernel matrix (*K*), which enables non-linear regression in a higher-dimensional feature space. This model considers that $K_{x_{i}x_{i}}^{\prime }=\exp\left(-\frac{h{∥x_{i}-x_{i}^{\prime }∥}^{2}}{p}\right)$, where *h* is a bandwidth parameter that controls the rate of decay between pairs of markers, and ${∥x_{i}-x_{i}^{\prime }∥}^{2}$ is the Euclidean distance between any two pairs of genotypes *i* and *i*′ normalized to range from 0 to 1. The RKHS model was implemented in the library BGLR (Pérez and de Los Campos, 2014) of R 3.6.1., which was run with 1,000,000 iterations, a burn-in period of 100,000, and a thin of 50.

## Bayesian Regularized Neural Network

The regularization process of BRNN was obtained by considering the weights (feature vector in the feature space, which represent the strength of connections between neurons) as random variables with a given prior distribution (defined below) according to Glória et al. (2016). In general, the structure of BRNN consists of three parts: (I) an input layer, which is given by genomic information of individuals (independent variables), (II) one hidden layer with n neurons that connect the input and output layers, and (III) an output layer with only one neuron that produces as output the prediction values of interest (Glória et al., 2016). The neurons allow the connection of the different layers inside a network, and the strength of the connection between neurons is called weight (Glória et al., 2016). The means of estimated weights measure the influence of the predictor variables on the response variable (learned information from training data). The posterior distribution for the weights from BRNN proposed by Gianola et al. (2011) can be accessed according to Bayes theorem:

$$\text{P}\left(\text{w}|\text{Y},\alpha ,\gamma ,\text{M}\right)=\frac{\text{P}\left(\text{Y}|\text{w},\gamma ,\text{M}\right)\text{P}\left(\text{w}|\alpha ,\text{M}\right)}{\text{P}\left(\text{Y}|\alpha ,\gamma ,\text{M}\right)}$$

where P(w|Y, α, γ, M) is the posterior distribution of the connection strengths, P(Y|w, γ, M) is the likelihood function, P(w|α, M) is the prior distribution for the weights vector, and P(Y|α, γ, M) is the marginal likelihood of the data; Y represents the observed data (markers genotypes matrix and the adjusted phenotypic values); *w* is the unknown weights vector; *M* denotes the architecture of the neural network used; α and γ are the regularization parameters that control the compensation between the smoothing of the network and goodness of fit (Gianola et al., 2011; Okut et al., 2011; Glória et al., 2016).

Ten BRNN-based architectures were tested, in which the following hyperparameters were considered to find the optimal architecture that increases the PA of genomic prediction: activation functions (i.e., linear: purelin; log-sigmoid: logsig; tangent sigmoid: tansig) and layers number (1–3 layers). The architectures tested with one layer (and one neuron) were brnn1, brnn2, and brnn3, in which each one considered the activation function purelin, logsig, and tansig, respectively. The architectures brnn4, brnn5, brnn6, brnn7, and brnn8 considered two layers, with two neurons and one neuron in each layer, respectively. The architectures brnn4, brnn5, and brnn6 consider in both layers a repetition of the activation function purelin, logsig, and tansig, respectively. Brnn7 and brnn8 used a combination of activation functions, in which brnn7 considered the activation functions tansig and purelin, in the first and second layer, respectively, while for brnn8, the activation functions logsig (layer 1) and purelin (layer 2) were considered. The architectures brnn9 and brnn10 considered three layers, a combination of the three activation functions and two, two, and one neuron in layers 1, 2, and 3, respectively. Brnn9 used tansig, logsig, and purelin activation functions in layers 1, 2, and 3, respectively, while brnn10 used logsig (layer 1), tansig (layer 2), and purelin (layer 3) activation functions (details about architectures are shown in Supplementary Table S1). All architectures of BRNN studied were fitted through the trainbr(x) function implemented in neural networks toolbox of Matlab 2019a (Beagle et al., 2019).

### Long Short-Term Memory Network (LSTM)

**FIGURE 1:**
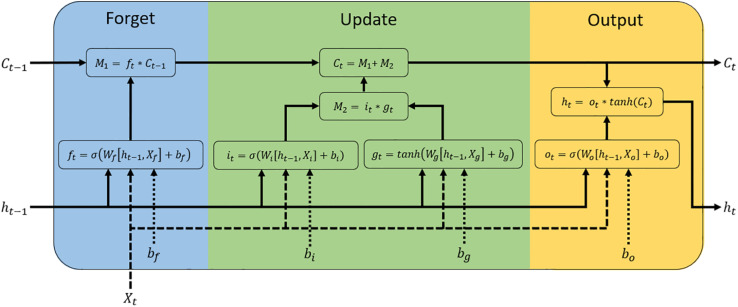
Diagram of a Long Short-Term Memory (LSTM) block. This block is a recurrently connected subnet that contains memory cell and gates functional modules. *X_t_*, *h*_*t*−1_, and *C*_*t*–1_ are the inputs of the LSTM unit, which correspond to the input of the current time step, the output from the previous LSTM unit, and the memory of the previous unit, respectively. C_*t*_ denotes the memory of the current unit and *h_t_* denotes the output of the current network (outputs of LSTM unit). The LSTM block is divided into three parts: gates forget (blue), update (green), and output the cell (yellow). Each part is composed of a sigmoid function (σ), which computes the gates activation function (f_*t*_: forget gate, i_*t*_: input gate, o_*t*_: output gate) from input weights (*WX*_*t*_), recurrent weights (*Wh*_*t*−1_), and bias (*b*). The update and output parts use a hyperbolic tangent (Tanh) function to calculate the input update (*g_t_*) and the memory of the current unit (*h_t_*), respectively.

LSTMs are a special kind of recurrent neural network (RNN) designed to learn long-term dependencies (Pouladi et al., 2015; Pérez-Enciso and Zingaretti, 2019). These networks are one of the most popular methods of RNN for their favorable convergence properties, adding additional interactions per module (or cell) and allowing one to overcome the vanishing gradient problem, which is a difficult task in RNN (Pouladi et al., 2015; Le et al., 2019). A typical LSTM network is composed of memory blocks called cells (Sak et al., 2014; Hua et al., 2019; Le et al., 2019). These cells are a recurrently connected subnet that contains memory cell in charge of remembering the temporal state of the neural network and gates responsible for controlling the flow of information and avoid the long-term dependency problem (Figure 1).

The first step for constructing a LSTM network is to determine what kind of information is not required and will be removed from the memory cell state. This process is implemented by a sigmoid function (called forget gate), which is determined by a vector with values ranging from 0 to 1, corresponding to each number in the cell state (Le et al., 2019). This function takes the part from the old output (*h*_*t*–1_) at time *t* − 1, and the current input (*X_t_*) at time *t*, for calculating the components that control the cell state and hidden state of the layer:

$$\text{C}L_{t}=\sigma \left({\text{W}}_{CL}\left[{\text{h}}_{t-1},{\text{X}}_{CL}\right]+{\text{b}}_{CL}\right)$$

where *CL* represents the components of the LSTM layer (input gate [*i_t_*], forget gate [*f_t_*], and output gate [*o_t_*]), σ is the sigmoid function, while *W* and *b* are the weight matrices and bias (respectively). The learnable weights and bias of the LSTM layer are the input weights *W* (Input Weights), the recurrent weights *R* (Recurrent Weights; associated with *h*_*t*–1_), and the bias *b* (Bias). These matrices are concatenations of their weights and bias in each component, respectively (Beagle et al., 2019).

$$W=\left[\begin{array}{c} W_{i}\\ W_{f}\\ \begin{array}{c} W_{g}\\ W_{o} \end{array} \end{array}\right],R=\left[\begin{array}{c} R_{i}\\ R_{f}\\ \begin{array}{c} R_{g}\\ R_{o} \end{array} \end{array}\right],b=\left[\begin{array}{c} b_{i}\\ b_{f}\\ \begin{array}{c} b_{g}\\ b_{o} \end{array} \end{array}\right]$$

where *i*, *f*, *g*, and *o* indicate the input gate, forget gate, cell candidate, and output gate, respectively.

The next step is divided into two parts: (1) deciding whether the new information should be updated or ignored (0 or 1) through a sigmoid layer (*i_t_*), and (2) deciding the level of importance (−1 to 1) of values that passed through a hyperbolic tangent (*tanh*) layer (*g_t_*). Next, these two parts are combined to trigger an update to the memory cell state, wherein this new memory is then added to old memory *C*_*t*–1_ (at time *t* − 1) resulting in *C_t_* (at time *t*).

$${\text{g}}_{t}=\text{tanh}\left({\text{W}}_{n}\left[{\text{h}}_{t-1},{\text{X}}_{t}\right]+{\text{b}}_{n}\right)$$

$${\text{C}}_{t}={\text{f}}_{t}*{\text{C}}_{t-1}+{\text{i}}_{t}*{\text{g}}_{t}$$

The last step is to decide the output values, which is performed by multiplication between the value obtained from the output of the sigmoid layer and the new values created by the hyperbolic tangent (Tanh) layer from the cell state (*C_t_*) (ranging between −1 and 1).

$${\text{h}}_{t}={\text{O}}_{t}\text{tanh}\left({\text{C}}_{t}\right)$$

The LSTM layer, by default, uses the Tanh function to compute the state activation function. Pouladi et al. (2015) replaced the Tanh function by a Rectified Linear Units (ReLU) learning strategy, for genotype imputation and phenotype sequences prediction. This study shows that the ReLU methods have a better performance in training (less error) compared to other LSTM models and better phenotype prediction compared to the results of the sparse partial least squares method. In this model, the recurrent weight matrix is initialized to an identity matrix and the biases are set to zero (Pouladi et al., 2015). Considering the previous observations, six LSTM architectures were tested with different activation functions (Tanh or ReLU) and subsets of the training set to evaluate the gradient of the loss function and update of the weights (mini-batch). The architectures lstm1, lstm3, and lstm5 used the ReLU activation function, while for lstm2, lstm4, and lstm6, the Tanh activation function was used. The mini-batch considered 10, 50, and 100% of the training dataset. In this sense, lstm1 and lstm2 used a mini-batch of 10%, while for lstm3 and lstm4, a mini-batch of 50% was considered. For lstm5 and lstm6, a mini-batch of 100% was used (details about architectures are shown in Supplementary Table S2). All architectures of LSTM networks were implemented in Matlab (Beagle et al., 2019) (details about scripts are shown in Supplementary Data Sheet S1). LSTM is named in other parts of the manuscript as “Deep Learning” (DL).

### Estimates of SNP Effects From ML Models

Glória et al. (2016) were the first to empirically test the BRNN model for estimating marker effects (considering from one to three layers) through methods proposed by Dimopoulos et al. (1995) and Goh (1995), which are based on partitioning the connection weights to determine the relative importance of the SNP markers and the sensitivity of the network for each SNP, respectively. However, models of DL typically consider multiple hidden layers and different types of layers (e.g., fully connected or normalization layers) for reducing the regression errors (Khaki and Wang, 2019), which prevents the use of methods proposed by Goh and Dimopoulos. In this sense, Wang et al. (2012) proposed an estimation of SNP effects by using the vector of genotype effects (or breeding values, **a**), the diagonal matrix of weights for variances of SNPs (*D*), and a matrix relating genotypes of each locus (*Z*), which represent substitution effects for each marker locus (coding {AA, Aa, aa} as {0, 1, 2}). It is assumed that the vector of genotype effects (or breeding values) is a function of SNP effects (VanRaden, 2008; Strandén and Garrick, 2009; VanRaden et al., 2009; Misztal et al., 2012; Wang et al., 2012), such that:

a=Zu

where *u* is a vector of SNP marker effects. Therefore, considering the equation of Strandén and Garrick (2009), the SNP effects is given by:

u^=DZ[ZDZ]′-1′a^

Wang et al. (2012) created an iterative algorithm for the estimation of *D* from $\hat{a}$ and *Z*. In the present study, *D* was estimated for three iterations of this algorithm, which proceeded as follows:

1. ${\hat{u}}_{t}=D_{t}Z^{\prime }{\left[ZD_{t}Z^{\prime }\right]}^{-1}$ where $\hat{a}$ is the genotype effects (or breeding values),
  *D*_0_ is the identity matrix, when *t* = 0.
2. $D_{t+1}^{*}={\hat{u}}_{i_{t}}^{2}2p_{i}\left(1-p_{i}\right)$ where *i* is the *i*th SNP of *Z* matrix
3. $D_{t+1}=\frac{tr\left(D_{t0}\right)}{tr\left(D_{t+1}^{*}\right)}D_{t+1}^{*}$
4. *t* = *t* + 1
5. Exit if *t*> 3, else loop to step 2.

Finally, the vector of prediction of SNP effects is given by ${\hat{u}}_{t}$.

In each model (Bayesian alphabet models, GBLUP, RKHS, BRNN, and LSTM), the PA was measured as the average of Pearson correlation coefficient between observed and predicted phenotypes in the validation set. The GP methods evaluated in this study were assessed by 50 cycles of cross-validation, in which the dataset was divided randomly into two independent training (90%) and validation (10%) groups. In the dataset of *Z. mays*, 290 genotypes were randomly selected as training dataset at each cycle of cross-validation, and the remainder 32 genotypes were used as validation samples. Similarly, in *E. globulus*, each cycle of cross-validation was performed considering 581 trees (randomly selected) as training dataset and the remainder 65 trees as validation set. The Tukey–Kramer test was performed to compare the PA values for each trait among the evaluated models.

## Results

### The Importance of Hyperparameters in DL and BRNN Architectures

Tables 1, 2 show the PA, obtained by cross-validation, of the hyperparameter combinations in each architecture tested for DL (LSTM) and BRNN, respectively. The lstm5 network had the highest PA values among all the DL networks tested. Moreover, lstm5 was statistically different from all models for ASI (environment CAM, maize) and WD (eucalypt). In the DL models, the use of ReLU as the activation function in the architectures lstm3 (mini-batch = 50%) and lstm5 (mini-batch = 100%) was more efficient in terms of PA values. In the architectures with smaller mini-batches (10%), ReLU was the function with higher PA values in the eucalypt population, while the hyperbolic tangent (Tanh) function was better in maize (Table 1). This result indicates that the ReLU function is more efficient in terms of PA than Tanh when the mini-batch size increased. This finding may be due to the fact that the optimization and backpropagation of the error by the ReLU function is more efficient when gradient estimates are less noisy and have larger partial data (large mini-batch), whereas Tanh is more efficient when subsets of the training set are smaller (small mini-batch) and gradient estimates are noisier (Masters and Luschi, 2018; Thafar et al., 2019). On the other hand, methods with mini-batch of 100% (lstm5 and lstm6) were the most efficient in terms of the computational time required for the genomic prediction of genotype effects in the cross-validations, performing up to four times faster than the mini-batches of 10% (Supplementary Table S3).

**TABLE 1:** Predictive ability of complex traits in maize (FF, female flowering; MF, male flowering; ASI, anthesis-silking interval) and eucalypt (WD, pilodyn penetration; ST, stem straightness; BQ, branch quality; TH, tree height; DBH, diameter at breast height) for six deep learning models, considering different hyperparameters: activation function (Rectified Linear Units: lstm1, lstm3, and lstm5; hyperbolic tangent: lstm2, lstm4, and lstm6) and mini-batch (10%: lstm1 and lstm2, 50%: lstm3 and lstm4, 100%: lstm5 and lstm6).

Model	*Zea mays*						*Eucalyptus globulus*				
	FF		MF		ASI		WD	ST	BQ	TH	DBH
	Sabaudia	Cambira	Sabaudia	Cambira	Sabaudia	Cambira					
lstm1	0.533^*b*^	0.724^*b*^	0.623^*a*^	0.764^*ab*^	0.455^*a*^	0.552^*b*^	0.317^*d*^	0.469^*b*^	0.422^*b*^	0.368^*cd*^	0.377^*d*^
lstm2	0.545^*ab*^	0.740^*ab*^	0.631^*a*^	0.757^*b*^	0.492^*a*^	0.548^*bc*^	0.271^*e*^	0.416^*c*^	0.382^*c*^	0.350^*d*^	0.369^*d*^
lstm3	0.558^*ab*^	0.742^*ab*^	0.639^*a*^	0.763^*ab*^	0.486^*a*^	0.561^*b*^	0.395^*b*^	0.481^*b*^	0.388^*c*^	0.423^*b*^	0.472^*b*^
lstm4	0.539^*ab*^	0.737^*ab*^	0.628^*a*^	0.751^*b*^	0.475^*a*^	0.506^*c*^	0.365^*c*^	0.345^*d*^	0.343^*d*^	0.408^*bc*^	0.404^*cd*^
lstm5	0.565^*a*^	0.751^*a*^	0.639^*a*^	0.776^*a*^	0.528^*a*^	0.610^*a*^	0.471^*a*^	0.557^*a*^	0.460^*a*^	0.496^*a*^	0.556^*a*^
lstm6	0.558^*ab*^	0.730^*ab*^	0.627^*a*^	0.765^*ab*^	0.488^*a*^	0.537^*bc*^	0.408^*b*^	0.558^*a*^	0.436^*ab*^	0.474^*a*^	0.452^*bc*^

*Predictive ability values followed by a common letter are not significantly different according to the Tukey–Kramer test at a level of significance of 0.01.*

**TABLE 2:** Predictive ability of complex traits in maize (FF, female flowering; MF, male flowering; ASI, anthesis-silking interval) and eucalypt (WD, pilodyn penetration; ST, stem straightness; BQ, branch quality; TH, tree height; DBH, diameter at breast height) for Bayesian regularized neural network models, considering different hyperparameters: activation function (pureline: brnn1, brnn4, brnn7, brnn8, brnn9, and brnn10; logsig: brnn2, brnn5, brnn8, brnn9, and brnn10; tansig: brnn3, brnn6, brnn7, brnn9, and brnn10) and number of layers (one layer: brnn1, brnn2, and brnn3, two layers: brnn4, brnn5, brnn6m brnn7, and brnn8, three layers: brnn9, and brnn10).

Model	*Zea mays*						*Eucalyptus globulus*				
	FF		MF		ASI		WD	ST	BQ	TH	DBH
	Sabaudia	Cambira	Sabaudia	Cambira	Sabaudia	Cambira					
brnn1	0.481^*ab*^	0.617^*ab*^	0.548^*ab*^	0.709^*a*^	0.447^*a*^	0.461^*a*^	0.454^*a*^	0.469^*a*^	0.419^*a*^	0.491^*a*^	0.490^*a*^
brnn2	0.410^*c*^	0.548^*cd*^	0.436^*c*^	0.609^*c*^	0.272^*b*^	0.230^*c*^	0.211^*f*^	0.333^*de*^	0.349^*d*^	0.374^*bc*^	0.399^*c*^
brnn3	0.307^*d*^	0.294^*e*^	0.311^*d*^	0.673^*ab*^	0.337^*ab*^	0.453^*a*^	0.271^*e*^	0.398^*c*^	0.311^*e*^	0.349^*c*^	0.390^*c*^
brnn4	0.486^*a*^	0.652^*a*^	0.584^*a*^	0.710^*a*^	0.423^*a*^	0.460^*a*^	0.466^*a*^	0.459^*a*^	0.412^*ab*^	0.504^*a*^	0.501^*a*^
brnn5	0.444^*abc*^	0.607^*ab*^	0.543^*ab*^	0.672^*ab*^	0.333^*ab*^	0.379^*ab*^	0.223^*f*^	0.412^*c*^	0.391^*bc*^	0.410^*b*^	0.463^*ab*^
brnn6	0.413^*bc*^	0.593^*bc*^	0.439^*c*^	0.618^*c*^	0.406^*a*^	0.459^*a*^	0.371^*d*^	0.427^*bc*^	0.374^*c*^	0.380^*bc*^	0.409^*c*^
brnn7	0.434^*abc*^	0.604^*ab*^	0.533^*ab*^	0.322^*d*^	0.413^*a*^	0.407^*ab*^	0.385^*cd*^	0.345^*d*^	0.208^*g*^	0.378^*bc*^	0.400^*c*^
brnn8	0.444^*abc*^	0.596^*bc*^	0.497^*b*^	0.641^*bc*^	0.415^*a*^	0.286^*bc*^	0.397^*bc*^	0.315^*e*^	0.260^*f*^	0.409^*b*^	0.432^*bc*^
brnn9	0.440^*abc*^	0.581^*bcd*^	0.521^*b*^	0.637^*bc*^	0.407^*a*^	0.454^*a*^	0.407^*b*^	0.422^*bc*^	0.374^*c*^	0.375^*bc*^	0.403^*c*^
brnn10	0.426^*abc*^	0.532^*d*^	0.520^*b*^	0.647^*bc*^	0.39^*ab*^	0.416^*a*^	0.231^*f*^	0.443^*ab*^	0.222^*g*^	0.301^*d*^	0.253^*d*^

*Predictive ability values followed by a common letter are not significantly different according to the Tukey–Kramer test at a level of significance of 0.01.*

In BRNN architectures, the brnn1 and brnn4 networks showed the best PA. In the flowering traits, the PA of the brnn4 network was the highest within Bayesian models, while that in wood-related traits, the highest PA was in brnn1. Although the PA values of the brnn1 model were slightly lower than brnn4 in flowering traits of maize, the predictions of both networks were very competitive, and these evidenced no significant differences (Table 2). Notably, brnn1 was the most efficient in terms of the computational time required in comparison with brnn4, with differences of up to 12.4 h (Supplementary Table S4). This result is due to fact that the architectures that used one layer converged faster than architectures with two or three layers. On the other hand, the activation functions in the Bayesian architectures are related to the PA values, since the architectures with one or two layers were more efficient (in terms of PA) when the purelin activation functions were used and showed lower PA when the logsig function was used.

The above results indicated that lstm5 and brnn1 were the most efficient architectures in the prediction of the study traits, due to their high PA values and low computational time required. Therefore, these architectures were selected to compare PA values with Bayes A, Bayes B, Bayes Cπ, BL, GBLUP, RKHS, and BRR in the prediction of the study traits.

### Prediction Ability for Complex Traits in Maize and Eucalypt

The predictive abilities of complex traits for each of the nine methods: BL, BRR, Bayes A, Bayes B, Bayes Cπ, GBLUP, RKHS, BRNN (brnn1), and DL (lstm5) in maize and eucalypt populations are shown in Tables 3, 4, respectively. PA values for flowering-related traits varied between 0.42 for ASI (environment SAB) and 0.78 for MF (environment CAM) for RKHS and DL, respectively (Table 4). Consistently, DL had the highest predictive ability (PA = ∼0.56) for ST and DBH in the eucalypt population, while the BL showed the lowest PA value in BQ (PA = 0.06). In general, there were no important differences among Bayesian linear methods, GBLUP and RKHS, except in computational time required, where GBLUP was the less time-consuming model (Supplementary Tables S5, S6). On the other hand, the DL (lstm5) model had the highest PA for all study traits, in comparison to all models tested. Moreover, lstm5 network had PA values significantly higher than the other models of genomic prediction in almost all traits (Tables 3, 4).

**TABLE 3:** Estimates of predictive ability of complex traits for different genomic models assessed in 6 years old eucalypt trees.

Model/traits	WD	ST	BQ	TH	DBH
Bayes A	0.267^*e*^	0.376^*d*^	0.216^*d*^	0.304^*c*^	0.352^*e*^
Bayes B	0.295^*d*^	0.518^*b*^	0.128^*g*^	0.319^*c*^	0.341^*e*^
Bayes Cπ	0.455^*b*^	0.544^*ab*^	0.162^*f*^	0.441^*b*^	0.394^*d*^
BL	0.301^*cd*^	0.200^*e*^	0.056^*h*^	0.204^*d*^	0.169^*g*^
BRR	0.321^*c*^	0.481^*c*^	0.309^*c*^	0.303^*c*^	0.444^*c*^
GBLUP	0.187^*g*^	0.226^*e*^	0.142^*g*^	0.159^*e*^	0.220^*f*^
RKHS	0.223^*f*^	0.225^*e*^	0.180^*e*^	0.197^*d*^	0.230^*f*^
BRNN	0.454^*b*^	0.469^*c*^	0.419^*b*^	0.491^*a*^	0.490^*b*^
DL	0.471^*a*^	0.557^*a*^	0.460^*a*^	0.496^*a*^	0.556^*a*^
${\hat{h}}^{2}\left(\text{SE}\right)$*	0.09 (0.05)	0.01 (0.03)	0.05 (0.04)	0.04 (0.04)	0.01 (0.03)

The study traits were pilodyn penetration (WD), stem straightness (ST), branch quality (BQ), tree height (TH), and diameter at breast height (DBH) of eucalypt trees. Predictive ability values followed by a common letter are not significantly different according to the Tukey–Kramer test at a level of significance of 0.01. *Narrow-sense heritability. SE, standard error.

**TABLE 4:** Estimates of predictive ability of complex traits for different genomic models assessed in maize inbred lines.

Model/traits	FF		MF		ASI	
	Sabaudia	Cambira	Sabaudia	Cambira	Sabaudia	Cambira
Bayes A	0.512^*bc*^	0.635^*b*^	0.592^*b*^	0.652^*c*^	0.464^*b*^	0.510^*c*^
Bayes B	0.499^*bc*^	0.633^*b*^	0.567^*bc*^	0.661^*c*^	0.462^*b*^	0.550^*b*^
Bayes Cπ	0.487^*c*^	0.648^*b*^	0.586^*bc*^	0.644^*c*^	0.469^*ab*^	0.533^*bc*^
BL	0.498^*bc*^	0.624^*b*^	0.561^*bc*^	0.664^*c*^	0.527^*a*^	0.540^*bc*^
BRR	0.511^*bc*^	0.617^*b*^	0.594^*b*^	0.660^*c*^	0.479^*ab*^	0.543^*bc*^
GBLUP	0.531^*ab*^	0.558^*c*^	0.563^*bc*^	0.645^*c*^	0.429^*b*^	0.454^*d*^
RKHS	0.526^*b*^	0.560^*c*^	0.590^*b*^	0.667^*c*^	0.421^*b*^	0.469^*d*^
BRNN	0.481^*c*^	0.617^*b*^	0.548^*c*^	0.709^*b*^	0.447^*b*^	0.461^*d*^
DL	0.565^*a*^	0.751^*a*^	0.639^*a*^	0.776^*a*^	0.528^*a*^	0.610^*a*^
*H*^2^(SE)	0.606 (0.12)	0.847 (0.08)	0.614 (0.12)	0.778 (0.1)	0.287 (0.1)	0.295 (0.1)
${\hat{h}}_{a}^{2}\left(\text{SE}\right)$	0.208 (0.43)	0.506 (0.47)	0.206 (0.43)	0.758 (0.54)	0.253 (0.14)	0.274 (0.12)
${\hat{h}}_{d}^{2}\left(\text{SE}\right)$	0.398 (0.48)	0.341 (0.49)	0.408 (0.48)	0.020 (0.57)	0.034 (0.12)	0.021 (0.1)

*The study traits were female flowering (FF), male flowering (MF), and anthesis-silking interval (ASI) of maize plants. Predictive ability values followed by a common letter are not significantly different according to the Tukey–Kramer test at a level of significance of 0.01. H^2^ is the broad-sense heritability; ${\hat{h}}_{a}^{2}$ and ${\hat{h}}_{d}^{2}$ are the additive and dominance ratios, respectively. SE, standard error.*

ML methods (BRNN and DL) required less time in comparison to the other genomic prediction methods assessed (Bayes A, Bayes B, Bayes Cπ, BRR, BL, and RKHS). This may be due to the combination of hyperparameters used in both architectures, which increase the PA of genomic prediction and may increase computational efficiency. Moreover, the Bayes A, Bayes B, Bayes Cπ, BRR, BL, and RKHS algorithms are implemented via Markov chain Monte Carlo (MCMC) for sampling from the posterior distribution of SNP effects, which is computationally expensive (Pérez and de los Campos, 2013; Wang et al., 2015). In this regard, GBLUP was the computationally most efficient model, running each iteration in ∼1 min (Supplementary Tables S5, S6). However, this model was not better than the DL method in terms of the PA for all traits.

### Estimates of SNP Effects in DL Model

**FIGURE 2:**
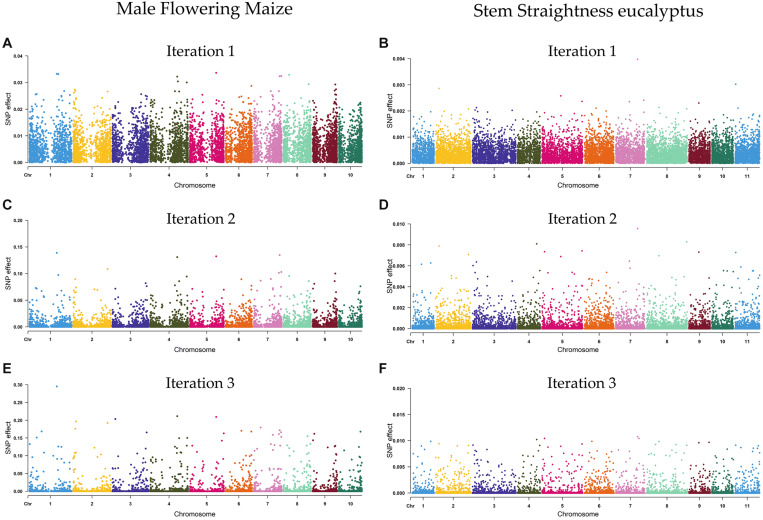
Estimates of marker effects obtained using the deep learning model for male flowering (MF) of maize **(A,C,E)** and stem straightness (ST) of 6 years old eucalypt trees **(B,D,F)**. Three iterations of the algorithm of Wang et al. (2012) are shown.

The SNP marker effects of the DL (lstm5) model, estimated using the iterative algorithm of Wang et al. (2012), are shown in Supplementary Data Sheet S2. The estimate of the first iteration was similar to those obtained by the BRR model, while the marker effect estimates of the second and third iterations were not similar to BRR (Supplementary Figures S1, S2), since the marker effects were re-estimated in each iteration, reducing or increasing their values (Supplementary Figure S3). As an example, Figure 2 shows the estimates of marker effects (in absolute terms) for male flowering of maize plants and stem straightness of eucalypt trees, estimated using the DL (lstm5) method (estimates of marker effects for all traits are presented in Supplementary Figures S4, S5). For ST, the marker effect estimates varied from 1.2 × 10^–8^ to 4.0 × 10^–3^, with an average of 3.8 × 10^–4^, considering the first iteration of the algorithm, whereas for MF, the marker effects varied from 3.4 × 10^–7^ to 3.4 × 10^–2^, with a mean of 5.9 × 10^–3^.

The use of iterative algorithm (Wang et al., 2012) showed that the markers with small effects were reduced in the second iteration (Figure 2), while the markers with large effects were increased even more in the second and third iteration. In fact, in the second and third iteration, SNPs with large effects increased approximately four and nine times their values (respectively) in both methods with respect to the first iteration. Therefore, the results from this algorithm can be used to map and identify QTLs, an aspect highlighted by Wang et al. (2012), that is, because the algorithm increases the differences between the SNPs with high and low effects, enhancing the visual interpretation of the plots (Figure 2). Based on this approach, SNPs with the greatest effect for MF of maize (SNP effects > 0.2) and for ST of eucalypt trees (SNP effects > 0.1) were arbitrarily considered as QTL. In this sense, four SNPs—on chromosomes 1, 3, 4, and 5—were associated with MF of maize plants, whereas five SNPs—on chromosomes 4, 5, 6, and 7—were associated with ST of eucalypt trees (Supplementary Data Sheet S3). Based on the physical position of maize reference genome^1^, 147 candidate genes were identified nearby (1 Mb) the SNPs with greatest effect. For *E. globulus*, considering the physical position of the reference genome of *E. grandis*^2^, a total of 277 candidate genes were identified nearby (1 Mb) the five SNPs with major effect (Supplementary Data Sheet S3).

## Discussion

Over the last several years, many approaches have been proposed to increase the prediction accuracy in GP studies, such as linear models of Bayesian alphabet (Pérez and de Los Campos, 2014; De Los Campos and Pérez-Rodríguez, 2016). However, few approaches have included non-parametric approaches and non-linear functions. In this study, two ML-based approaches that implement non-parametric methods and numerous non-linear activation functions were used (Ho et al., 2019; Montesinos-López et al., 2019a, b; Pérez-Enciso and Zingaretti, 2019). The results of this study showed that the DL model had a higher PA than GBLUP, linear (Bayes A, Bayes B, Bayes Cπ, BRR, and BL) and non-linear (RKHS and BRNN) Bayesian regression models in the prediction of several complex traits in both breeding populations.

Bellot et al. (2018) developed genomic prediction models for human complex traits using UK Biobank data and found that the DL model was more competitive than the penalized linear methods. However, the predictive ability of DL was dependent on the study phenotype. Particularly, the results of this genomic prediction showed that DLs performed comparatively better as narrow-sense heritability decreased and the contribution of dominance increased. Similarly, Zingaretti et al. (2020) implemented GP in polyploid outcrossing species (i.e., strawberry and blueberry) and found that DL did not show clear advantages over the linear models BL and BRR, except when the non-additive effects (dominance or epistasis) were important. The authors also pointed out that the use of DL methods in GP of polyploid plants allows one to exploit its non-linearity, and it has less restrictive assumptions in comparison to traditional linear model-based methods. Moreover, polyploid plants might present higher degrees of complete and partial intra-locus interactions compared with diploid species (Zingaretti et al., 2020). These results are not in agreement with this study, in which the PA of DL did not evidence differences between the contributions of the additive or dominance effects (Table 4), since DL was the best method in the prediction (in terms of PA) of all traits. These findings may be due to the fact that Bellot et al. (2018) and Zingaretti et al. (2020) used the Convolutional Neural Networks, whereas in the present study, the LSTM method was used; however, we emphasize that other studies must be performed to corroborate this argument. Interestingly, Alves et al. (2020) compared the predictive performance of GBLUP with ANN method in simulated traits considering different levels of dominance effects. They found that ANN had a higher prediction accuracy compared with GBLUP for traits with moderate narrow-sense heritability (*h*^2^ = 0.30) and dominance effects of 0 or 0.15. In the present study, the DL approach outperformed GBLUP despite the low dominance effect (${\hat{h}}_{d}^{2}<$0.035; Table 4). This is indicative that DL is a promising alternative tool for GP independent on the contribution of additive and/or dominance genetic effects.

Several studies have reported that the combination of hyperparameters critically influences the predictive performance of the DL model, emphasizing the need to carefully optimize hyperparameters in the ML architectures (Glória et al., 2016; Bellot et al., 2018; Pérez-Enciso and Zingaretti, 2019; Zingaretti et al., 2020). Particularly, Glória et al. (2016) found that a simple architecture of BRNN outperformed other more complex architectures (by adding layers and/or more complex activation functions) in terms of PA. This finding implies that the complexity of neural networks provides a decrease in PA, increasing the standard error of prediction. In this study, 10 BRNN architectures were tested through the combination among the number of layers (1–3) and activation functions (purelin, logsig, and tansig). Similarly, the six architectures tested in the DL method correspond to the combination of the activation functions Tanh and ReLU, with different mini-batch sizes (10, 50, and 100%). A close association between the hyperparameter of activation function and the efficiency of the genomic prediction was observed in the BRRN model, as the higher PA values were observed in the brnn1 and brnn4, which had different numbers of layers (1 and 2, respectively), but the same activation functions (purelin). On the other hand, the poor performance of architectures with three layers could be due to an overfit to the training set, an aspect observed by Glória et al. (2016). However, it is worth noting that a combination of activation functions was used in the networks with three layers, and these activation functions were not repeated as in the networks with two layers. Therefore, the reduction in the PA values could be caused by the logsig and tansig functions. In spite of this, it was evidenced that brnn1 and brnn4 were very competitive, revealing that the increase in the number of layers did not present a significant increase in the PA. Moreover, the results showed that the increased number of layers was more expensive computationally (Supplementary Table S4).

The hyperparameters assessed in DL showed that the ReLU activation function was more efficient in terms of PA than the Tanh function when the mini-batch was larger. ReLU can represent a linear function and thus has the advantage of preserving the properties of linear and non-linear models, i.e., easy to optimize and backpropagate the error (Thafar et al., 2019). Furthermore, ReLU offers better performance and generalization in DL compared to the sigmoid and Tanh activation functions (Bouktif et al., 2018; Nwankpa et al., 2018). Hesamifard et al. (2017) observed that deep neural networks with the ReLU activation function had better performance in the classification of encrypted data compared to the sigmoid and Tanh functions. Similarly, Li et al. (2018) showed that the use of ReLU function greatly improves the performance over Tanh and sigmoid functions, in sequential MNIST classification and language modeling (using the character-level Penn Treebank dataset). On the other hand, a large mini-batch size was more efficient in terms of PA. Previous studies have indicated that a small mini-batch size achieves better training stability and generalization performance, while a larger mini-batch tends to have degradation in the quality of the model (Li et al., 2014; Keskar et al., 2016; Smith et al., 2017). Moreover, a smaller mini-batch increases the velocity of model updates and the efficient use of memory. Masters and Luschi (2018) showed that increasing the mini-batch size provides stable convergence and acceptable test performance. Furthermore, the training samples of the mini-batch are randomly drawn in every step, so the resulting gradients are less accurate and gradient estimates are noisier. In this study, the mini-batch did not affect the efficiency of genomic prediction; however, the time of processing was affected. In this sense, the increasing of mini-batch significantly reduced the training time (Supplementary Table S3), due to the fact that a large mini-batch size has fewer training processes in each epoch and takes less step to converge (Smith et al., 2017; Wang et al., 2017). Therefore, the best architecture in DL was lstm5 due to its efficiency in terms of PA and reduced computational times.

The Bayesian linear models did not show differences with BRNN in terms of PA. This was also noted by Glória et al. (2016) who did not observe differences among BRNN, BL, and RR-BLUP in the prediction of quantitative traits. On the other hand, DL had a better result than Bayesian models in terms of PA. In this sense, Liu and Wang (2017) found that DL outperformed traditional statistical models (RR-BLUP, BL, and Bayes A) in the genomic prediction of grain yield, in soybean, and stem height, in loblolly pine. In a GP study for meat tenderness, Lopes et al. (2020) found similar results to the current study, in which the DL model had higher PA than all models of the Bayesian alphabet (Bayes A, Bayes B, Bayes Cπ, BRR, and BL). Notably, in this study, ReLU was the best activation function used for training DL, because it is faster to learn than sigmoid and hyperbolic tangent functions, and it has better performance during the random grid search. The results of the present study indicated that DL can provide superior genomic predictions for quantitative traits, despite the relatively small sample sizes used (322 maize inbred lines and 646 half/full-sib progenies of eucalypt). The computational time required for the prediction analysis is also expected to be reduced in the DL method, as demonstrated in this study, since the hyperparameter selection can reduce the time of analysis and enhance the performance of genomic predictions.

From the genomic point of view, the molecular marker techniques used in this study present clear differences, which have been broadly studied and discussed (Pérez-Enciso et al., 2015; De Moraes et al., 2018). For example, De Moraes et al. (2018) pointed out that both techniques have differences in linkage disequilibrium patterns, MAF, missing data, and marker distribution. However, the results of their study, considering 13 wood quality and growth traits of *Eucalyptus* trees, demonstrated that both genotyping methods are equivalent in terms of PA in the GP models RR-BLUP and Bayes B. In this regard, the missing marker data and MAF are two major quality control factors in genome-wide studies (Ali et al., 2020). These factors along with the population size may affect the PA in GP models. In this sense, in studies of genomic prediction that consider a small population size, the PA values can be limited (Edwards et al., 2019). In the present study, we assessed two breeding populations that differ in population size, genotyping method (i.e., DNA chip array and GBS), the degree of missing marker data, and MAF values. Despite these differences, our results indicated that DL-based prediction models presented high PA values in both different breeding populations, indicating that this model can help to decrease phenotypic cost within breeding programs.

The present study leveraged the iterative algorithm of Wang et al. (2012) to estimate the marker effects of the DL method, which increased the differences between the markers with high and low effects, in each iteration. Therefore, the results of this iterative algorithm can be used to map and identify QTLs. In this sense, Wang et al. (2014) showed the accuracy of the iterative algorithm for the QTL identification in 6 weeks body weight in broiler chickens. In this study, four and five SNPs were considered as QTL due to its great effect on the expression of traits in maize (MF) and eucalypt (ST), respectively, according to Wang et al. (2012). Overall, 147 and 277 candidate genes were identified nearby the SNPs with a major effect for MF and ST, respectively. In eucalypt, chromosome 4 had three candidate genes (Eucgr.D02209, Eucgr.D02250, and Eucgr.D02208) that are described as RING zinc finger protein, which has been related to ST by Bartholomé et al. (2016) and Li et al. (2016) in radiata pine and maritime pine, respectively. Li et al. (2016) also identified a protein serine/threonine kinase associated with ST, which is in accordance with our finding, since four candidate genes (Eucgr.D02135, Eucgr.G02060, Eucgr.G02065, and Eucgr.G02273) presented this same description (Supplementary Data Sheet S3). On the other hand, candidate gene GRMZM2G111491 of maize is a homolog of AT4G29380 in *Arabidopsis thaliana*, which encodes phosphatidylinositol 3-kinase, a protein involved in the development and germination of pollen (Xu et al., 2011). Finally, it should be noted that the advantages of using this approach in the QTL identification include the possibility of using a complex model with single or multiple traits and a computational implementation that is fast and simple (Wang et al., 2012, 2014).

## Conclusion

Hyperparameter optimization is a fundamental step for successfully implementing a DL model. In this sense, the results of this study suggested that architectures with the activation function ReLU and a mini-batch of large size were the most optimal for the genomic prediction of complex traits in maize and eucalypt. Furthermore, our results showed that DL had a superior performance than GBLUP, Bayes A, Bayes B, Bayes Cπ, BRR, BL, RKHS, and BRNN. On the other hand, the iterative algorithm proposed by Wang et al. (2012) was first used in marker effect estimation from a DL model. This can be seen as a new approach for GWAS through DL, since it allows the identification of the most relevant genomic regions affecting the traits of interest. The results of this study confirm the importance of DL models in genome-wide studies and crop/tree improvement, which holds promise for accelerating breeding progress.

## Data Availability Statement

Publicly available datasets were analyzed in this study. These data can be found here: https://figshare.com/s/d5d83e58e4634532e706; https://figshare.com/s/5ecdc88adecedb 61e940.

## Author Contributions

FM-P, CM, CAS, and ATDAJ conceived the research plans. SA and J-TC performed the data curation. CM and FM-P analyzed the genomic data and wrote the first draft of the manuscript. FM-P, RIC-S, and CAS supervised the field experiments. FM-P, CM, SA, J-TC, and ATDAJ reviewed and edited the final version of the manuscript. All authors reviewed and approved the manuscript for publication.

## Conflict of Interest

The authors declare that the research was conducted in the absence of any commercial or financial relationships that could be construed as a potential conflict of interest.

## Footnotes

1 http://www.maizegdb.org//

2 https://phytozome.jgi.doe.gov//

## Supplementary Material

Supplementary Figures 1–5 Can be found here: https://figshare.com/s/6fcbf2f6e17e39c270b7.

Supplementary Tables 1–6 Can be found here: https://figshare.com/s/4c8cc56146632eb4170e.

Supplementary Data Sheets 1–3 Can be found here: https://figshare.com/s/5cd5e5e4eaeef55b721f.
